# British Medical Association

**Published:** 1905-03-11

**Authors:** 


### BRITISH MEDICAL ASSOCIATION.

### CONFERENCE ON HOSPITAL ADMINISTRATION BETWEEN REPRESENTATIVES OF THE ASSOCIATION AND OF HOSPITAL BOARDS.

A conference on hospital administration between the Hospitals Committee of the British Medical Association and representatives of hospitals, metropolitan and provincial, was held in the Board-room at the Metropolitan Asylums Board on Thursday, March 2nd, 1905.

Dr. William Collier, President of the British Medical Association, took the chair at 3 p.m.; the meeting was very largely attended.

The Chairman explained that the Hospitals Committee of the British Medical Association was apppointed some years ago in consequence of numerous letters that had appeared in the medical and lay Press calling attention to hospital abuse. The medical men on that Committee represented all parts of the British Isles, and included a very considerable number of medical men experienced in hospital management. The Committee had gone very carefully into the question of hospital abuse, and what reforms could be brought about in the management of hospitals. As a result they had brought forward a number of resolutions which had already been discussed with a few distinguished medical men. The Committee had invited a large number of laymen interested in hospital administration to a conference, in order to obtain their views on the subject. In taking a vote on the resolutions the Committee asked from the laymen their friendly criticism and advice, which would be very seriously considered by the Committee. It was proposed, if the principles embodied in the resolutions were accepted, to send them to the various hospitals, and it was hoped that in that way a public opinion in the matter of hospital reform would in time be created. If there was necessity for improvement, as medical men believed, it was felt that it was more likely to be brought about by friendly co-operation with the committees of management of the various hospitals, and medical men and the staffs of those hospitals. Hospital reform was desired to put an end to some of the abuses which existed. It would not benefit medical men alone; they felt very strongly indeed that it would benefit some of those who applied. If those people who could well afford to pay for medical advice could be prevented from attending the hospitals they would be taught very valuable lessons; that was to say, they would be taught the principle of self-reliance and habits of thrift. Speaking from 20 years' experience of hospitals he could say that the out-patients' departments were often so much congested, and the number of patients to be looked after was so large, that the medical officers could not give as much time as they felt they ought to give to individual patients. If the number applying were diminished the treatment of the others would be improved to a very great extent. In conclusion he explained that the members of the Hospitals Committee did not intend to vote. A vote would be taken in order to get a real indication of the feeling of the meeting on the matters under discussion.

Dr. Beverley (Chairman of the Hospitals Committee) submitted the resolutions for discussion. The first was as follows:

That inability to pay for adequate treatment shall be the consideration for the admission of all patients for hospital treatment.

Mr. Collins (Birmingham General Hospital) asked if the resolution was intended to include pauper cases, which should be treated by the Poor-law medical officers. As it stood, the resolution seemed to imply that the hospitals should treat all pauper cases.

Dr. Beverley said that the resolution as it stood covered Poor-law cases if they were suitable cases for hospital treatment, and were sent to the hospitals by Poor-law surgeons as such. He pointed out that the Committee did not suggest that all parish cases should be admitted, but only such as required special hospital treatment. It was far from the intention of the Committee that hospitals should in any way relieve Boards of Guardians from their responsibility, but he felt sure that it was the wish of all those who supported hospitals that if a parish or workhouse patient required special hospital treatment, surgical or medical, he should receive it whether he was a pauper or not.

Sir Henry Burdett (London) said that pauper cases had been admitted to all classes of hospitals throughout the country for many years, and the practice which was enforced by hospitals, large and small, from Guy's Hospital to cottage hospitals, was that the Poor-law authorities should pay so much per day during the residence of those cases in the hospitals. As it was the duty of the Boards of Guardians to provide adequate medical treatment to the fullest extent for all pauper cases, the Conference could not pass the resolution as it stood, and then pass the second resolution.

Dr. Pope suggested that a rider should be added to the effect that the resolution did not apply to Poor-law cases, and that they considered anything done for the Poor-law authorities should be paid for.

This suggestion was accepted by the Chairman of the Committee and the rider was ultimately appended to the resolution.

The resolution was then put and declared carried.

Dr. Beverley then brought up resolution 2 :

That no charge for the treatment of any patient shall be made.

Mr. Walter Baily (University College Hospital) inquired whether this resolution applied to paying wards in hospitals.

Dr. Beverley replied that the question of paying wards had not yet received the consideration of the Committee. There were various questions, of which this was one, which sooner or later must be referred to the Committee.

Sir Henry Burdett considered that the resolution was clearly in conflict with the first, unless it was proposed to exclude cottage hospitals altogether, and to admit by right to the wards of the hospital people who were attended in the ordinary course by their own medical man, but who, in the terms of the first resolution, were unable to pay for adequate treatment.

A lengthy discussion on the bearing of this resolution took place, and when the Chairman put the resolution to the meeting, 17 voted in favour and 28 against, and the resolution was declared lost.

Dr. Beverley then brought forward Resolution 3 :—

That the production of subscribers' letters shall cease to be compulsory, and that where possible the system shall be abolished.

He said that the Committee were aware that it could not be at once carried into effect, but it asked that the hospitals should entertain it and consider whether it was not possible to carry it into effect.

Dr. Beverley pointed out that the words "if possible" had purposely been inserted, as it was felt that, although it might not be possible in some places, it might be in many.

The Chairman then put the resolution, which was carried, only four voting against it.

Dr. Beverley then brought forward Resolutions 4, 5, 6, and 7, which were agreed to as follows, without discussion :—

4. That some means of investigation into the circumstances of the applicants for relief by means of an almoner or other agent shall be employed in all medical charities.
5. That the foregoing recommendations (Resolutions Nos. 1, 3, and 4) apply to both in-patients and out-patients.
6. That, except in emergencies, sufficient evidence shall be obtained on two points :—(*a*) That the patient is not in a position to pay for treatment; (*b*) that the case is, from a hospital point of view, suitable for treatment.
7. That all cases of serious accident and severe sudden illness shall be attended to on their first application, and, if deemed eligible for further treatment, shall be referred to the appropriate department of the hospital ; but, if ineligible, shall then be referred for treatment elsewhere.

Dr. Beverley then submitted Resolution 8 :—

8. That all cases of trivial accident or illness deemed ineligible for the out-patient department shall, after having been attended to, be referred for treatment elsewhere.

Hon. Sydney Holland thought that it might be said that a patient suffering from trivial accident or illness should not be treated at all in the out-patients' department.

Dr. Beverley pointed out that the Committee wished that every case should have first aid from the hospital.

After some further discussion as to the meaning of the words "attended to," the word "seen" was suggested in substitution therefor. This was accepted, and the resolution was put and adopted.

Dr. Beverley then brought forward Resolution 9 :—

9. That it is inexpedient that a patient be permitted to leave a hospital without having been seen by a registered medical practitioner.

Mr. Collins suggested that if taken literally this resolution could not be carried out in provincial hospitals ; but if it meant on the first visit he quite agreed with it.

The Chairman observed that that was the meaning of it.

Dr. Beverley said the Committee felt very strongly that every case which came to the hospital should, in the first instance, be seen by a qualified medical man—that is, one of the resident staff, who would be qualified.

After some further discussion, the resolution was put and adopted.

Sir Henry Burdett suggested the addition of the words "on the first visit," which Dr. Beverley agreed to.

Dr. Beverley then brought forward Resolutions 10 and 11 :—

10. That where possible the number of new cases to be seen on any one day by an honorary medical officer shall be limited. That special hospitals shall treat only those cases that come strictly within the scope of their work.
11. That in all hospitals there shall be an age limit for the retirement of the medical officers, which were put and agreed to.

Dr. Beverley then invited the conference to appoint twelve gentlemen specially competent to help the Hospitals Committee in carrying out these resolutions or amending them, and to bring them before the Boards of Management—both metropolitan and provincial. The Committee thought it was extremely important that these resolutions should not go forth to the hospitals as simply the resolutions of the British Medical Association or any body of medical men only. They would be far more likely to be carried out if they were known to be an expression of opinion from those who, as Mr. Sydney Holland put it, were perhaps more competent to judge of the management and organisation of hospitals than even medical men. If another conference was held in the future it would be public.

The following gentlemen were then proposed to serve on the Committee :—Sir Henry Burdett, K.C.B. ; Hon. Sydney Holland, Chairman London and Poplar Hospitals ; Mr. Howard Collins, House Governor Birmingham General Hospital ; Colonel Montefiore, Charity Organisation Society ; Mr. W. G. Bunn, Secretary Hospital Saturday Fund ; Sir Riley Lord, Newcastle-on-Tyne Infirmary ; Mr. Charles Lupton, Chairman Leeds General Infirmary ; Mr. Danvers

Power; Sir Charles Gilman, Norfolk and Norwich Hospital; Mr. Arthur E. Reade, Secretary Charing Cross Hospital; Mr. Herbert W. Mason, East Suffolk and Ipswich Hospital; Mr. W. G. Carnt, Derbyshire Royal Infirmary.

The meeting sanctioned power to add to its numbers.

On the motion of Dr. Beverley, seconded by Dr. Mary Thorne, a vote of thanks to those gentlemen who had attended the Conference was unanimously adopted, and the proceedings terminated.

